# On the brain struggles to recognize basic facial emotions with face masks: an fMRI study

**DOI:** 10.3389/fpsyg.2024.1339592

**Published:** 2024-01-26

**Authors:** Jubin Abutalebi, Federico Gallo, Davide Fedeli, Elise Houdayer, Federica Zangrillo, Daniele Emedoli, Alfio Spina, Camilla Bellini, Nicola Del Maschio, Sandro Iannaccone, Federica Alemanno

**Affiliations:** ^1^Centre for Neurolinguistics and Psycholinguistics (CNPL), Faculty of Psychology, Vita-Salute San Raffaele University, Milan, Italy; ^2^The Arctic University of Norway, Tromsø, Norway; ^3^Centre for Cognition and Decision Making, Institute for Cognitive Neuroscience, Higher School of Economics, National Research University, Moscow, Russia; ^4^Department of Neuroradiology, Fondazione IRCCS Istituto Neurologico Carlo Besta, Milan, Italy; ^5^Neuropsychology Service, Department of Rehabilitation and Functional Recovery, San Raffaele Scientific Institute, Vita-Salute San Raffaele University, Milan, Italy; ^6^Department of Neurosurgery and Gamma Knife Radiosurgery, San Raffaele Hospital, Milan, Italy

**Keywords:** facial emotion recognition, COVID-19, face mask, SARS-CoV-2, psychology, fMRI

## Abstract

**Introduction:**

The COVID-19 pandemic impacted public health and our lifestyles, leading to new social adaptations such as quarantine, social distancing, and facial masks. Face masks, covering extended facial zones, hamper our ability to extract relevant socio-emotional information from others’ faces. In this fMRI study, we investigated how face masks interfere with facial emotion recognition, focusing on brain responses and connectivity patterns as a function of the presence of a face mask.

**Methods:**

A total of 25 healthy participants (13F; mean age: 32.64 ± 7.24y; mean education: 18.28 ± 1.31y) were included. Participants underwent task-related fMRI during the presentation of images of faces expressing basic emotions (joy or fear versus neutral expression). Half of the faces were covered by a face mask. Subjects had to recognize the facial emotion (masked or unmasked). FMRI whole-brain and regions-of-interest analyses were performed, as well as psychophysiological interaction analysis (PPI).

**Results:**

Subjects recognized better and faster emotions on unmasked faces. FMRI analyses showed that masked faces induced a stronger activation of a right occipito-temporal cluster, including the fusiform gyrus and the occipital face area bilaterally. The same activation pattern was found for the neutral masked > neutral unmasked contrast. PPI analyses of the masked > unmasked contrast showed, in the right occipital face area, a stronger correlation with the left superior frontal gyrus, left precentral gyrus, left superior parietal lobe, and the right supramarginal gyrus.

**Discussion:**

Our study showed how our brain differentially struggles to recognize face-masked basic emotions, implementing more neural resources to correctly categorize those incomplete facial expressions.

## 1 Introduction

Since its spread in 2020, the COVID-19 pandemic has revolutionized our lives, and its impact is far from disappearing completely, even with the vaccination campaign raging on (The Lancet Microbe, 2021). Apart from social distancing, one of the most effective containment measures has been the adoption of face masks, which hide about 60–70% of the face (Carbon, 2020). The face is one of the most important means of social communication, both through verbal and non-verbal channels (Adolphs, 2002). In particular, the social relevance of the face has been related to its ability to express emotions through specific facial configurations. Facial emotion recognition is indeed crucial for the process of inferring our interlocutors’ emotional state (Adolphs, 2002). The adoption of facial masks has raised concerns about how these protective devices could interfere with facial emotion recognition during our social interactions (Carbon, 2020; Ruba and Pollak, 2020; Marini et al., 2021). For a successful facial emotion recognition, many individual facial features need to be extracted and then integrated into a unique percept (Ellison and Massaro, 1997). At the neural level, face perception is supported by a selective ventral occipito-temporal network that comprises the inferior occipital gyrus and the fusiform gyrus, known as the occipital face area (OFA) (Pitcher et al., 2007) and the fusiform face area (FFA) (Kanwisher et al., 1997), respectively. Indeed, the ventral stream is more involved in facial expression processing than the dorsal stream and includes the right inferior occipital gyrus (containing the right OFA), the left middle occipital gyrus, left FFA, and the right inferior frontal gyrus (Liu et al., 2021). The OFA is generally considered as the first stage of face processing as it responds selectively to single facial features (Pitcher et al., 2011b), which are then integrated into a unique facial representation in the FFA (Pitcher et al., 2007). Thus, these two brain areas are considered to be involved in the processing of the invariant aspects of a face, such as its canonical T configuration, which allows for the recognition of each individual as unique (Haxby et al., 2002). However, a face also displays dynamic features, such as eye gaze, mouthing, and facial expressions. These aspects are processed separately in different subdivisions of the superior temporal sulcus (STS) (Schobert et al., 2018) and then transmitted to other brain regions (e.g., the amygdala) to extract their socio-emotional relevance during interactions with others (Haxby et al., 2002; Gan et al., 2022).

The covering of important facial features by face masks may hamper face processing ability, leading to misinterpretations of the emotion expressed and to misunderstandings in social contexts. Previous studies have shown that masking heavily affected emotion recognition, reducing the recognizability of facial expressions (Proverbio and Cerri, 2022; Rinck et al., 2022) and emotion recognition accuracy (Carbon, 2020; Ruba and Pollak, 2020; Calbi et al., 2021; Grundmann et al., 2021; Marini et al., 2021). A previous EEG study investigated the cerebral activity related to the recognition of six masked/unmasked facial expressions by using event-related potentials (ERPs) analyses (Żochowska et al., 2022; Proverbio et al., 2023). The results indicated increased neuronal activity during face-masked emotion recognition. They also showed that face masking was more detrimental to sadness, fear, and disgust than positive emotions, such as happiness. The authors suggested that processing of faces with surgical-like masks might require an amplified attentional process. Indeed, hiding crucial facial characteristics with face masks may induce a re-organization of the neural resources involved in face processing. A better understanding of such neuronal plasticity induced by face masks is still needed. In this study, we aimed to further investigate how face masks may influence brain responses and connectivity during facial emotion recognition. Given the novelty of mask use in everyday life, little is known about how the brain accommodates the recognition of facial expressions covered by masks. We used functional magnetic resonance imaging (fMRI) to determine whether this process would differ from normal “uncovered” facial emotion recognition (Wegrzyn et al., 2015).

## 2 Materials and methods

### 2.1 Participants

A total of 25 right-handed healthy volunteers (13F; mean age: 32.64 ± 7.24 y; mean education: 18.28 ± 1.31 y) were included in this study. The study was approved by the Ethics Committee of the Ospedale San Raffaele (Milan), and all participants gave their oral and written informed consent in accordance with the Declaration of Helsinki.

### 2.2 Facial emotions recognition task and analyses

**Figure 1:**
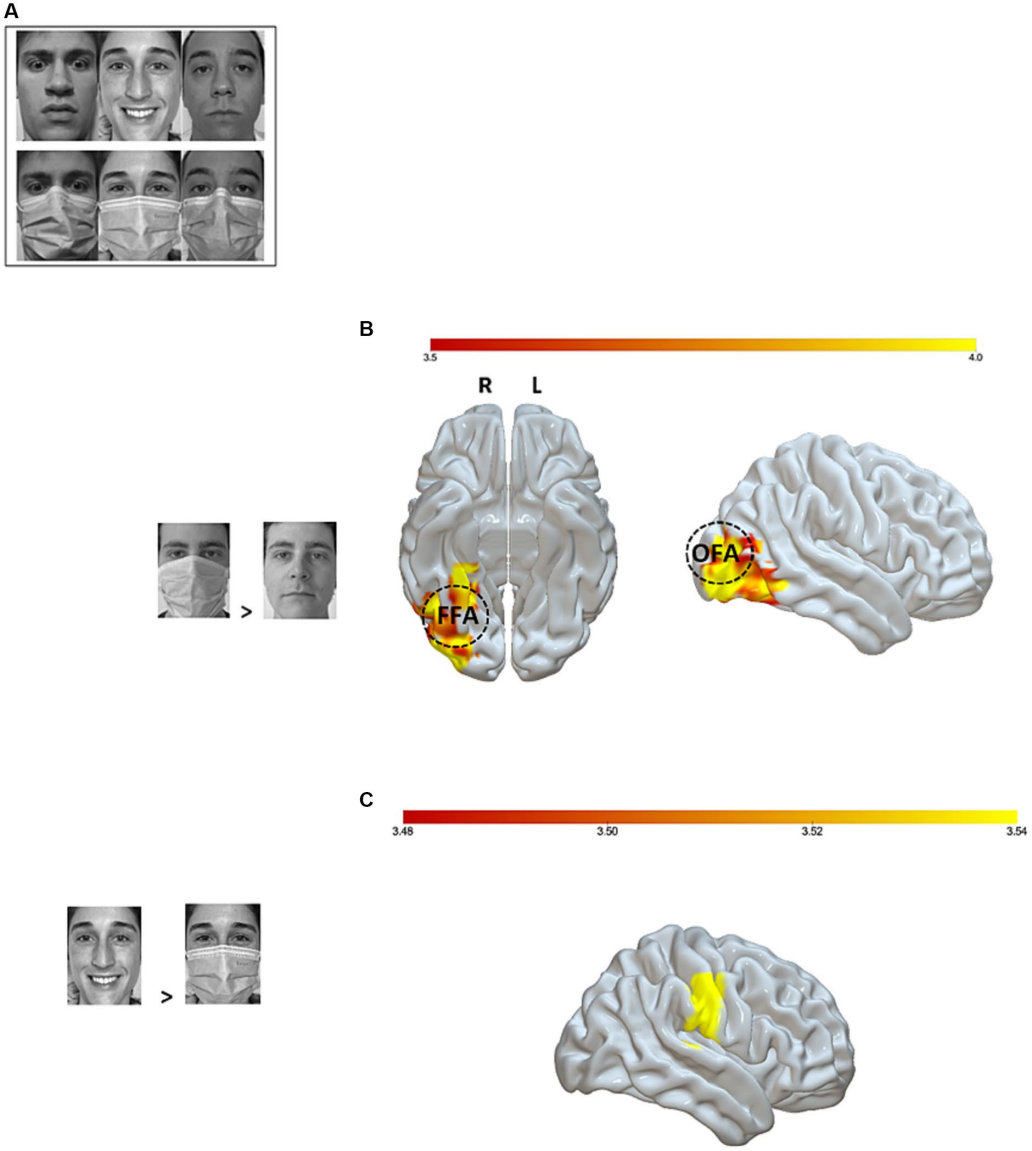
fMRI setup and analyses. **(A)** Examples of experimental stimuli; **(B)** Significant brain activations (*p*-uncorrected < 0.001 voxel level and *p*-FWE < 0.05 cluster level) for the masked > unmasked contrast. The right fusiform face area (rFFA) is represented at MNI coordinates *x* = 40, *y* = −55, *z* = −12 and the right occipital face area (rOFA) at MNI coordinates *x* = 39, *y* = −79, *z* = −6, 10 mm diameter. **(C)** Significant brain activation (*p*-uncorrected <0.001 voxel level and *p*-FWE < 0.05 cluster level) for the joy unmasked > joy masked contrast. Color bar represents *t*-values. OFA, Occipital Face Area; FFA, Fusiform Face Area; L, Left; R, Right.

During the fMRI session, participants were shown black-and-white pictures of faces expressing a basic emotion (joy or fear), along with a neutral control condition. In all, 36 pictures were used and presented twice to the participants during each block for a total of three blocks. Thus, each participant was exposed to a total of 216 pictures. Half of the target faces in each condition were covered with a surgical mask (Figure 1A). Participants were instructed to press a response button on an MRI-compatible response box corresponding to the emotion expressed by each face, whether masked or not masked, in order to capture differential brain responses.

The stimuli were collected by the experimenters by taking close-up photographs of 5 young Caucasian male adults and 6 young Caucasian female adults expressing six emotions (fear, joy, disgust, surprise, sadness, anger) or a neutral pose, each with and without a surgical mask on. This picture database underwent ratings by 25 independent participants who had to assign one of the seven conditions to each masked and unmasked picture. Based on these ratings, we selected as experimental stimuli the single negative (i.e., fear) and positive (i.e., joy) emotions with the highest level of correct assignments, together with the neutral pose, and the 3 male and 3 female participants whose sets of facial expressions received the highest level of correct assignments. This procedure resulted in a set of 36 experimental stimuli: 6 individuals expressing three emotions (joy, fear, neutral) with two mask conditions (masked, unmasked). Faces were projected onto a panel that was reflected inside the scanner by a mirror glass. The 36 images were shown to all participants at 4:3 ratio, in black and white, on a white background. During a de-briefing session, all the participants were asked to evaluate the emotion associated with each of the experimental pictures via an online questionnaire. No picture was removed due to poor ratings (< 60%).

Pictures were presented to the participants for 2000 ms. When the stimulus disappeared, a question mark was displayed on the screen for 500 ms. The question mark prompted the participants to choose the appropriate emotion (joy, fear, or neutral). This stimulus was then followed by a blank screen that lasted for the remaining duration of the inter-stimulus interval (ISI, approximately 2,398 ms). Therefore, participants had almost 3 s to rate each picture before the beginning of the following trial. The Presentation software^1^ was used to present stimuli and collect participants’ responses.

To explore the main effects of both mask condition (i.e., mask and unmask) and emotion category (i.e., joy, fear, and neutral), as well as their interaction, on the participants’ accuracy rates and reaction times (RTs), a generalized linear mixed model was computed with the *glmer* function in the “lme4” package (Bates et al., 2015). Response correctness (for accuracy analyses) or RTs for hit responses were entered into the model as dependent variables. Participants were modeled as random intercept. Mask condition and emotion category were modeled as fixed effects, and each was tested for its significance by comparing a model in which the fixed term of interest was present against a model in which it was not included (i.e., likelihood ratio test). A predictor was retained only when its inclusion determined a significant increase in explained variance. In case of a significant interaction, all the lower-order terms involved were retained. *Post hoc* comparisons were run with the emmeans package. Data were considered significant when *p* < 0.05. Statistical analyses were run on R software (version 4.3.0) (R Development Core Team, 2015).

### 2.3 fMRI recording and analyses

MR images were acquired with a 3Tesla Philips Ingenia CX MR system (Philips HealthCare, Best, Netherlands) equipped with a 32-channels SENSE head coil. A fast-speed echo-planar imaging (EPI) sequence was used to acquire functional scans (echo time [TE] = 33 ms; repetition time [TR] = 2000 ms; flip angle [FA] = 85°; number of volumes per run = 199; field of view [FOV] = 240 mm; matrix size = 80 × 80; 35 axial slices per volume; slice thickness = 3 mm; interslice gap = 0.75 mm; voxel size = 3 × 3 × 3.75 mm^3^; phase-encoding direction [PE] = A/P; whole-brain coverage). A total of 10 dummy scans preceded each run to optimize the EPI image signal. Pre-processing was run using the default pre-processing batch available in spm12. In particular, prior to undergoing pre-processing, the origin of each T1w image was manually aligned to the anterior commissure–posterior commissure (AC-PC) line. Then, MR images were subjected to both temporal and spatial pre-processing steps. First, functional images were slice-time corrected to the first slice to correct for differences in slice acquisition times, realigned to the first volume, and unwarped to remove movement artifacts. For each participant, functional volumes were checked for excessive head motion (>2 mm). No participant was excluded due to excessive head motion. T1w images were segmented into different tissue classes (i.e., grey matter, white matter, cerebro-spinal fluid, bone, soft tissues, and air), bias-corrected for intensity inhomogeneities, and spatially normalized. Then, the bias-corrected T1w images were skull-stripped using the “Image Calculator” SPM function, entered as reference image to co-register the mean realigned functional image of each participant, and normalized to the standard Montréal Neurological Institute (MNI) template. After normalization, functional volumes were resampled to 2 × 2 × 2 mm^3^ voxels and smoothed with a 6 mm^3^ full width at half-maximum (FWHM) Gaussian kernel to minimize inter-subject variability. After being pre-processed, functional data were analyzed at the whole-brain level using SPM12 (Wellcome Department of Cognitive Neurology, London, UK) (SPM v6906) by means of a random-effects model implemented with a two-level summary statistic approach. In the first-level analysis, evoked responses for the six experimental conditions were entered into a general linear model (GLM) and modeled with the canonical hemodynamic function (HRF). Realignment parameters were entered as nuisance covariates in the first-level analyses. Moreover, temporal autocorrelation was accounted for with an AR (1) regression algorithm. A 128 s high-pass filter was imposed, which removed slow signal chains with a longer period. A set of Student’s *t*-test linear contrasts were defined to use the estimated con-images at the second statistical level. At the second level of analysis, the contrast images obtained at the single-subject level were used to compute one-sample t-tests, assessing their significance at the group level. Voxel-wise whole-brain analysis was performed with cluster-level multiple comparison correction. The statistical threshold was set at cluster level at *p* < 0.05 family-wise error corrected (FWE) and at voxel level at *p* < 0.001.

We then narrowed the focus of our analyses on regions of interest (ROIs) known to play a key role in facial emotion recognition and facial recognition *per se.* Through the MarsBaR toolbox for SPM (Brett et al., 2002), we defined as ROIs the OFA and the FFA, known to underpin facial processing (Kanwisher et al., 1997; Grill-Spector et al., 2017). The WFU_pickatlas toolbox of the Automatic Anatomical Labeling atlas (AAL2) (Tzourio-Mazoyer et al., 2002) was used to generate the anatomical ROIs for the bilateral amygdala, widely considered as critical for facial emotion recognition (Adolphs et al., 1994; Geissberger et al., 2020). Only whole-brain significant contrasts underwent ROI-based analysis. The statistical threshold was set at *p* < 0.05.

Finally, we performed a psychophysiological interaction analysis (PPI) by means of the gPPI toolbox for SPM8^2^ in order to explore whether the presence of a facial mask changed the connectivity pattern between those regions and other brain areas during facial emotion recognition. Indeed, generalized PPI (gPPI) analysis offers the opportunity to understand how brain regions interact in a task-dependent manner in block or event-related task designs with two or more experimental conditions. As ROIs, we selected once again the bilateral OFA, FFA, and amygdala. For each ROI, a GLM was performed, including as regressors the BOLD signal extracted from that ROI (i.e., the physiological effect), the six experimental conditions (i.e., the psychological variables), and the element-by-element product of these two variables (i.e., the psychophysiological interaction). The statistical threshold was set at cluster level at *p* < 0.05 family-wise error corrected (FWE) and at voxel level at *p* < 0.001.

## 3 Results

### 3.1 Behavioral results

A significant mask condition × emotion category interaction on the participants’ accuracy rates was observed (χ^2^ = 17.498, value of *p* = 0.0002). *Post hoc* comparisons revealed that the probability of correct response was lower for fear mask than fear unmask faces (odds.ratio = 0.264, st.err. = 0.054, z.ratio = −6.567, value of *p* < 0.0001), for joy mask than joy unmask faces (odd.s ratio = 0.169, st.err. = 0.032, z.ratio = −9.279, value of *p* <0.0001), and for neutral mask than neutral unmask faces (odds.ratio = 0.545, st.err. = 0.111, z.ratio = −2.979, value of *p* = 0.034). Moreover, the probability of correct responses was lower for both fear mask (odds.ratio = 0.587, st.err. = 0.098, z.ratio = −3.203, value of *p* = 0.017) and joy mask (odds.ratio = 0.345, st.err. = 0.055, z.ratio = −6.721, value of *p* < 0.0001) than neutral mask faces. Lastly, the probability of correct responses was higher for fear mask than joy mask faces (odds.ratio = 1.699, st.err. = 0.242, z.ratio = 3.732, value of *p* = 0.0026). No such differences between emotion categories were observed for the unmask condition (*p* > 0.1).

Regarding the reaction times’ analyses, a significant mask condition x emotion category interaction on the participants’ reaction times was observed (χ^2^ = 10.238, value of *p* = 0.006). *Post hoc* comparisons showed that the participants took longer to respond to fear mask vs. fear unmask faces (beta = 0.104, st.err. = 0.025, t.ratio = 4.149, value of *p* = 0.0005), to joy mask vs. joy unmask faces (beta = 0.213, st.err. = 0.026, t.ratio = 8.221, value of *p* < 0.0001), and to neutral mask vs. neutral unmask faces (beta = 0.123, st.err. = 0.025, t.ratio = 4.977, value of *p* < 0.0001). Moreover, reaction times were longer for fear unmask vs. joy unmask faces (beta = 0.127, st.err. = 0.024, t.ratio = 5.228, value of *p* < 0.0001) and for neutral unmask vs. joy unmask faces (beta = 0.116, st.err. = 0.025, t.ratio = 4.278, value of *p* < 0.0001). No such differences between emotion categories were found for mask faces (*p* > 0.1). All behavioral results are reported in Table 1.

**Table 1:** Descriptive statistics for reaction times and accuracy ratings.

	RTs (s)	Accuracy (%)
	Mean ± SD	Mean ± SD
Fear mask	1.87 ± 0.89	83.19 ± 37.43
Fear unmask	1.75 ± 0.91	94.30 ± 23.20
Joy mask	1.84 ± 0.87	75.44 ± 43.08
Joy unmask	1.63 ± 0.97	93.86 ± 24.02
Neutral mask	1.89 ± 0.91	88.89 ± 31.45
Neutral unmask	1.75 ± 0.92	93.27 ± 25.06

Means and standard deviations are reported separately for each emotion category (i.e., fear, joy, and neutral) and masking condition (i.e., mask and unmask faces). Reaction times (RTs) are reported in seconds (s), whereas accuracy ratings are reported in percentages (%).

### 3.2 fMRI results

#### 3.2.1 Whole-brain analysis

At the neural level, whole-brain fMRI analyses revealed a significantly stronger activation of a right occipito-temporal cluster, including the fusiform gyrus, known to govern facial recognition (Rossion et al., 2000; Gerrits et al., 2019; Rigon et al., 2019), when participants saw masked versus unmasked faces (Figure 1B). These results were corroborated when investigating the same contrast in the neutral condition. On the contrary, the unmasked joyful expressions evoked a stronger activity in the right post-central gyrus with respect to their masked counterparts (Figure 1C). The neutral vs. emotional expressions comparison showed a bilateral response in the inferior subdivision of the lateral occipital cortex, along with an increased activation of the right fronto-parietal regions. The same contrast in the masked condition revealed a pronounced activity in occipital and frontal areas. These significant contrasts are shown in Table 2. Moreover, given the exploratory nature of this study (*n* = 25 participants), we double-ran the whole-brain analyses with a less stringent threshold (*p* < 0.05 FDR-corrected) and have reported the data in the Supplementary Table S1.

**Table 2:** Contrasts analyses.

**Contrast**	**Hemisphere**	**Region (Harvard-Oxford)**	***k* (mm** ^**3**^ **)**	***Z-*score**	**Cluster P FWE**	***x***	***y***	***z***
**Masked > Unmasked**	R	Lateral occipital cortex (inferior subdivision)	588	4.669	< 0.001	36	−80	8
	R	Occipito-temporal fusiform cortex	101	4.140	0.045	28	−40	−22
	R	Inferior temporal gyrus (temporo-occipital part)	147	4.015	0.008	48	−58	−8
**Neutral Masked > Neutral Unmasked**	R	Occipito-temporal fusiform cortex	109	5.207	0.049	26	−50	−12
	R	Inferior temporal gyrus (temporo-occipital part)	171	4.905	0.006	50	−58	−8
	R	Lateral occipital cortex (inferior subdivision)	393	4.638	<0.001	42	−76	8
**Joy Unmasked > Joy Masked**	R	Right post-central gyrus	232	4.18	0.029	56	−16	44
**Neutral > Emotion**	R	Lateral occipital cortex (inferior subdivision)	998	5.20	<0.001	36	−86	−8
	L	Lateral occipital cortex (inferior subdivision)	289	4.66	<0.001	−36	−88	−12
	R	Frontal pole	157	4.50	0.005	48	42	18
	R	Precentral gyrus	127	4.18	0.014	44	4	28
	R	Superior parietal lobule	175	5.09	0.002	32	−52	44
**Neutral Masked > Emotion Masked**	R	Occipital Pole	192	4.26	0.002	26	−92	−8
	L	Occipital Pole	114	3.83	0.03	−22	−94	−6
	R	Medial Frontal Gyrus	325	4.17	< 0.001	34	6	60

Significance threshold was set at voxel *p*-uncorrected <0.001 and cluster *p*-FWE-corrected <0.05. Coordinates are in MNI space. Only one local maximum per significant cluster is reported. Only significant contrasts are reported. R, right hemisphere; L, left hemisphere; FWE, family-wise error. Region labels are from Harvard-Oxford Atlas.

#### 3.2.2 ROI-based analysis

The ROI-based analyses on the ventral occipito-temporal regions supported the whole-brain analyses’ results: a significantly stronger activation emerged in the right FFA (*t* = 2.67, *p* = 0.006) and in the OFA bilaterally (rOFA: *t* = 4.72, *p* < 0.0001; lOFA: *t* = 3.16, *p* = 0.002) for the masked > unmasked contrast. The same activation pattern was found also for the neutral masked > neutral unmasked contrast (rFFA: *t* = 3.32, *p* = 0.0014; rOFA: *t* = 3.22, *p* = 0.0018; lOFA: *t* = 1.90, *p* = 0.034). The increased bilateral response of these regions remained significant also in the neutral vs. emotional expressions contrast, regardless of the presence of a face mask (rFFA: *t* = 3.43, *p* = 0.001; lFFA: *t* = 2.10, *p* = 0.023; rOFA: *t* = 5.00, *p* < 0.0001; lOFA: *t* = 2.91, *p* = 0.003). On the other hand, the unmasked > masked contrast revealed a greater response of the right amygdala (*t* = 1.97, *p* = 0.03). Interestingly, the right amygdala showed an increased activity also for the joy unmasked > joy masked contrast (*t* = 1.87, *p* = 0.037). Lastly, emotional faces (i.e., both joy and fear expressions), as compared to neutral ones, were associated with an increased response in the left amygdala (*t* = 2.06, *p* = 0.025), irrespective of the mask condition.

#### 3.2.3 Psychophysiological interaction analysis

**Figure 2:**
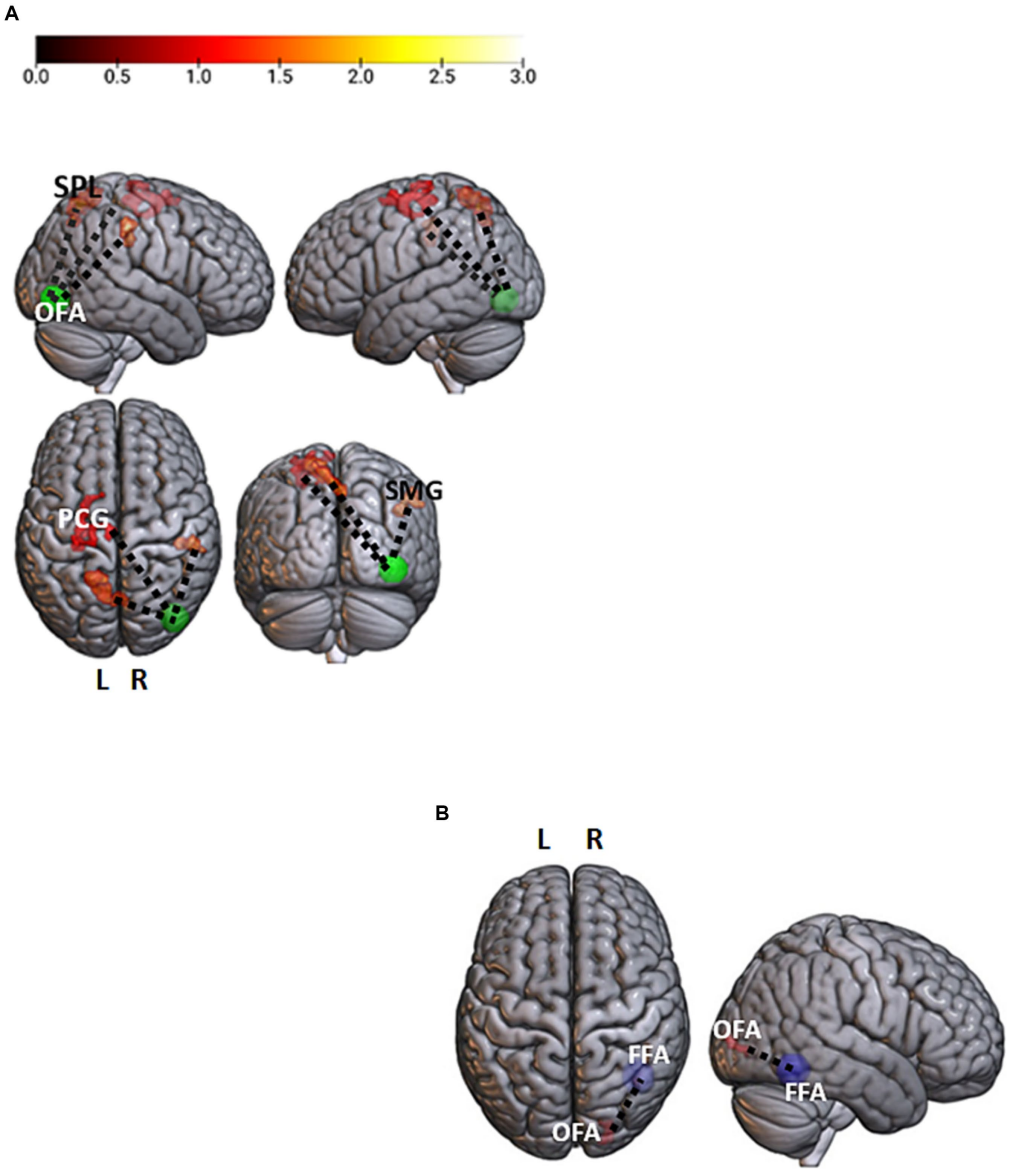
Facial emotions and neutral contrasts analyses. **(A)** Brain regions showing a significant stronger association with the right occipital face area (rOFA, p-uncorrected <0.001 voxel level and p-FWE < 0.05 cluster level) for the masked > unmasked contrast. The rOFA is represented at MNI coordinates *x* = 39, *y* = −79, *z* = −6, 10 mm diameter. Color bar represents *t*-values. **(B)** A significant stronger correlation between the right fusiform face area (rFFA; *x* = 40, *y* = −55, *z* = −12) and the homolateral OFA (rOFA; *x* = 39, *y* = −79, *z* = −6) for the neutral unmasked > neutral masked contrast (*p*-uncorrected <0.001 voxel level and *p*-FWE < 0.05 cluster level). OFA, Occipital Face Area; FFA, Fusiform Face Area; PCG, PreCentral Gyrus; SPL, Superior Parietal Lobe; SMG, Supramarginal Gyrus; L, Left; R, Right.

In the masked > unmasked contrast, the right OFA showed a stronger correlation with a set of bilateral fronto-parietal regions, the left superior frontal gyrus (*t* = 4.9; *p* < 0.001), the left precentral gyrus (*t* = 4.41; *p* < 0.001), the left superior parietal lobe (*t* = 5.89; *p* < 0.001), and the right supramarginal gyrus (*t* = 6.2; *p* < 0.001) (Figure 2A). The unmasked > masked contrast in the neutral condition led to a greater correlation between the right FFA and the right inferior occipital gyrus (*t* = 6.11; *p* < 0.001) (Figure 2B).

## 4 Discussion

The behavioral and fMRI results reported in this study suggest that the use of face masks may tax our ability, speed, and neural effort in facial emotion recognition.

Our behavioral results, in line with the recent literature, suggest that the use of masks reduces our ability to recognize facial expressions, both in terms of accuracy and response times, regardless of the emotion expressed (Carbon, 2020; Ruba and Pollak, 2020; Marini et al., 2021; Wong and Estudillo, 2022). These data suggest that the area of the face between the chin and cheekbones, hidden by the surgical mask, is equally essential for the recognition of both emotional expressions and a neutral face (Beaudry et al., 2013). However, accuracy data seem to indicate the greater importance of the mouth for the identification of joy compared to fear or neutral expressions (Wegrzyn et al., 2017).

FMRI analyses showed that the ventral occipito-temporal face recognition areas were more active when a mask was covering the target faces. Such a result might have been expected since masks hide important key features of the visual image of a face, forcing the observer to interpret the underlying facial expression only on the basis of the visible characteristics. As the OFA responds preferentially to single features, in particular to those conveyed by the eye and eyebrow regions (Arcurio et al., 2012), which are not covered by face masks, its greater activation for masked faces might indicate a higher reliance on the upper face region during facial emotion recognition. Consistent with this interpretation, highly relevant facial features led to a greater activation of the FFA (Nestor et al., 2008). These data suggest that the eye and forehead regions have a crucial role in the interpretation of masked facial expressions. Thus, the greater activation of both occipito-temporal areas may be expressive of a brain compensatory strategy in the case of the lack of crucial facial features.

Additionally, as revealed by the PPI analysis, the presence of a face mask was associated with a stronger correlation between the right OFA and fronto-parietal regions, supporting functions involved in the top–down regulation of emotional stimuli, such as episodic memory (Fletcher et al., 1995), visual working memory (Haxby et al., 2000), and attention (Wojciulik and Kanwisher, 1999). This result suggests that, for a masked face, the OFA (in which a visual structural analysis of the face is performed) needs a deeper communication with higher-order brain areas to correctly categorize and interpret the emotion it conveys. Moreover, unmasked neutral faces elicit a stronger communication between the right FFA and the homolateral OFA.

These findings indicate that the right OFA and the right FFA, independently, respond more strongly to masked neutral faces than to unmasked neutral faces, pointing to the need for more neural resources to correctly categorize and interpret these stimuli. Similarly, previous EEG studies showed larger N170 during the emotion recognition of masked (vs. unmasked) faces, suggesting that the processing of faces with surgical-like masks might require an amplified attentional process (Prete et al., 2022; Żochowska et al., 2022; Proverbio et al., 2023). One might hypothesize that the higher activations of the two ventral occipito-temporal regions support, in a compensatory manner, a feature-based processing of the face in order to identify its underlying expression on the basis of the configuration of the eyes and the forehead. On the other hand, the psychophysiological results for neutral unmasked faces, as compared to neutral masked, are consistent with the view of the OFA as the first stage of face processing, in which single facial features are extracted and then transferred to the FFA, which computes a facial *Gestalt*, essential to individual identity recognition (Pitcher et al., 2011a). Indeed, the processing of a neutral face might stop at this stage as there are no emotional meanings to extract. However, neutral expressions, relative to emotional ones, evoke greater involvement of the fronto-parietal regions, responsible for a higher-order facial processing (Atkinson and Adolphs, 2011). Our results are consistent with those of Carvajal et al. (2013), who observed that neutral expressions, with respect to emotional ones, led to the activation of a more complex representation requiring a more effortful cognitive processing.

At the behavioral level, we observed that neutral masked faces were recognized more accurately than joy or fear masked faces. Numerous behavioral studies showed that the recognition – in terms of accuracy – of masked neutral facial expressions, as compared to other emotional expressions (e.g., fear, happiness, sadness), is not so strongly affected by a face mask (Carbon, 2020; Carbon et al., 2022; Kim et al., 2022; Huc et al., 2023), paralleling our findings. This could be due to the fact that the area between the cheeks and the chin, i.e., the facial region typically covered by a face mask, may not be as necessary for the recognition of a neutral facial expression as it is for other emotional expressions, such as joy and fear. This may be best exemplified by how we instantly recognize faces conveying joy because of the contour of the mouth and lips forming a ´smile.´ This may also further corroborate recent findings that face masks result in more emotional expressions being confused with neutral ones (Carbon, 2020; Kim et al., 2022). Despite masked neutral expressions being better recognized at the behavioral level, we observed that they led to greater activity in the occipito-temporal and frontal brain areas as compared to masked emotional expressions. This increased fronto-occipito-temporal activation might reflect a more elaborate cognitive processing (Carvajal et al., 2013), not required for “basic” emotional expressions such as fear or joy. We suggest that, as aforementioned, since a neutral expression is not associated with a particular change in the whole facial configuration, it requires a more demanding cognitive effort, both visually and cognitively, to be correctly recognized with respect to joyful and fearful expressions, whose correspondent mouth and eye movements are extremely specific. These observations coming from previous literature provide motivation for the apparently contradictory pattern of the results observed, in which, at the behavioral level, masked neutral faces were better recognized, while at the neural level, masked neutral faces led to greater activity in circuits involved in a more effortful cognitive processing. Behaviorally, the greater recruitment of neural resources for the recognition of masked faces was paralleled by less accurate and quick performances when participants were struggling to classify emotions from a masked face. This observation confirms our neural findings that the face area between the chin and the cheekbones, covered by a face mask, is highly important for the recognition of both emotional expressions.

Interestingly, emotional faces (i.e., both joy and fear expressions), as compared to neutral ones, were associated with an increased response in the left amygdala, irrespective of the mask condition. As reported by many studies (Kensinger and Schacter, 2006; Lewis et al., 2007; Colibazzi et al., 2010; Tamietto and de Gelder, 2010), the amygdala plays a crucial role in regulating the processing of emotionally arousing stimuli. Gläscher and Adolphs (2003) attested to a predominant role of the left amygdala in the ability to detect stimulus arousal. In this study, we found that our emotional faces were more arousing than neutral expressions, even when the same expressions were half-covered by a surgical-like mask. We also showed a decreased amygdalar activation for masked joyful expressions as compared to unmasked ones, whereas not a such decrease was evident in the fear condition. This could be due to the fact that the key facial feature of joy expression is the smile (Leppänen and Hietanen, 2007; Bombari et al., 2013), which is covered by face masks, while fear is mostly expressed through the eyes and forehead (Wegrzyn et al., 2017), which are spared by mask use. Consistently with this interpretation, at the whole-brain level an unmasked joyful expression, with respect to a masked one, was found to lead to a greater involvement of the mouth area in the somatotopic representation of the secondary somatosensory cortex (SII), leading specifically to an enhanced activity in the dorso-caudal subdivision of the right post-central gyrus, which is devoted to the sensorimotor processing of lip movements (Penfield and Rasmussen, 1950; Grabski et al., 2012). Thus, the greater response of this region for an expression of joy might reflect the underlying processing of the structural changes that occur in the other’s face when the mouth widens into a smile.

To conclude, our results have highlighted the adaptability of the human neural systems underlying facial expression recognition in the condition brought forth by the spread of COVID-19. One limitation of this study was the utilization of static faces. Indeed, in our daily life and social interactions, we are usually exposed to dynamic faces. Further studies should thus confirm such results by including dynamic faces in the testing setup. Although face masks hamper our ability to interpret facial expressions of emotions and determine a more effortful neural processing of faces, one can rely, in daily life, upon other important cues allowing a correct identification of interlocutors’ emotional states, thus compensating for the lack of important facial information covered by face masks.

## Data availability statement

The original contributions presented in the study are included in the article/Supplementary material, further inquiries can be directed to the corresponding author.

## Ethics statement

The studies involving humans were approved by Comitato etico Ospedale San Raffaele, Milan, Italy. The studies were conducted in accordance with the local legislation and institutional requirements. The participants provided their written informed consent to participate in this study. Written informed consent was obtained from the individual(s) for the publication of any identifiable images or data included in this article.

## Author contributions

JA: Conceptualization, Methodology, Writing – review & editing, Investigation, Writing – original draft. FG: Investigation, Writing – original draft, Data curation, Formal analysis. DF: Data curation, Investigation, Methodology, Writing – original draft. EH: Data curation, Writing – review & editing. FZ: Writing – review & editing. DE: Writing – review & editing. AS: Formal analysis, Writing – review & editing. CB: Formal analysis, Investigation, Writing – original draft. ND: Investigation, Supervision, Writing – original draft. SI: Funding acquisition, Resources, Supervision, Visualization, Writing – review & editing. FA: Conceptualization, Methodology, Resources, Writing – review & editing.

## Footnotes

^1^ https://neurobs.com/

^2^ http://www.nitrc.org/projects/gppi
