# Supplementary material for: On the brain struggles to recognize basic facial emotions with face masks: an fMRI study
Source: Front Psychol. 2024 Jan 26;15:1339592. doi: 10.3389/fpsyg.2024.1339592 (PMC10858449; doi:10.3389/fpsyg.2024.1339592)
Supplement: Supplementary file 1 [file Table_1.docx]

**Table S1: Contrasts analyses.** Significance threshold was set at voxel p-uncorrected <0.001 and cluster p-FDR-corrected < 0.05. Coordinates are in MNI space. Only one local maximum per significant cluster is reported. Only significant contrasts are reported. R, right hemisphere; L, left hemisphere; FWE, family-wise error. Region labels are from Harvard-Oxford Atlas.

| **Contrast** | **Hemisphere** | | **Region (Harvard-Oxford)** | **k (mm^3^)** | **Z score** | **cluster P FDR** | **x** | **y** | **z** |
| --- | --- | --- | --- | --- | --- | --- | --- | --- | --- |
| **Masked > Unmasked** | R | Lateral Occipital Cortex (inferior subdivision) | | 3983 | 6.05 | < 0.001 | 36 | -80 | 8 |
|  | R | Occipito-Temporal Fusiform Cortex | | 1395 | 4.29 | 0.009 | 28 | -40 | -22 |
|  | R | Insula | | 1741 | 4.75 | < 0.001 | 28 | 28 | 6 |
|  | L | Inferior Occipital Cortex | | 1744 | 4.36 | < 0.001 | -36 | -74 | -4 |
|  | L | Inferior Frontal Gyrus pars orbitalis | | 2224 | 4.32 | < 0.001 | -42 | 32 | -6 |
| **Neutral Masked > Neutral Unmasked** | R | Occipito-Temporal Fusiform Cortex | | 4099 | 7.20 | < 0.001 | 26 | -50 | -12 |
|  | L | Middle Occipital Cortex | | 3030 | 4.58 | < 0.001 | -38 | -84 | 8 |
| **Joy Unmasked > Joy Masked** | R | Right Post-Central Gyrus | | 25541 | 5.13 | < 0.001 | 56 | -16 | 44 |
| **Neutral > Emotion** | R | Lateral Occipital Cortex (inferior subdivision) | | 4526 | 7.18 | < 0.001 | 36 | -86 | -8 |
|  | L | Lateral Occipital Cortex (inferior subdivision) | | 2130 | 6.02 | < 0.001 | -36 | -88 | -12 |
|  | R | Frontal Pole | | 3896 | 5.71 | < 0.001 | 48 | 42 | 18 |
|  | R | Supplementary Motor Area | | 6166 | 5.38 | < 0.001 | 6 | 6 | 56 |
| **Neutral Masked > Emotion Masked** | R | Occipital Pole | | 11526 | 5.28 | < 0.001 | 26 | -92 | -8 |
|  | L | Occipital Pole | | 2753 | 4.56 | < 0.001 | -22 | -94 | -6 |
|  | L | Inferior Frontal Cortex pars opercularis | | 4680 | 4.42 | < 0.001 | -56 | 12 | 30 |
